# Adjunctive dexamethasone treatment in adults with listeria monocytogenes meningitis: a prospective nationwide cohort study

**DOI:** 10.1016/j.eclinm.2023.101922

**Published:** 2023-03-24

**Authors:** Matthijs C. Brouwer, Diederik van de Beek

**Affiliations:** Department of Neurology, Amsterdam UMC, University of Amsterdam, Amsterdam Neuroscience, Amsterdam, the Netherlands

**Keywords:** Listeria monocytogenes, Bacterial meningitis, Dexamethasone, Prognosis

## Abstract

**Background:**

A French cohort study described a detrimental effect of adjunctive dexamethasone treatment in listeria meningitis. Based on these results guidelines recommend not to use dexamethasone if *L. monocytogenes* is suspected or stop dexamethasone when the pathogen is detected. We studied clinical characteristics, treatment regimens and outcome of adults with *Listeria monocytogenes* meningitis in a nationwide cohort study on bacterial meningitis.

**Methods:**

We prospectively assessed adults with community-acquired *L. monocytogenes* meningitis in the Netherlands between Jan 1, 2006, and July 1, 2022. We identified independent predictors for an unfavourable outcome (Glasgow Outcome Scale score 1 to 4) and mortality by logistic regression.

**Findings:**

162 out of 2664 episodes (6%) of community-acquired bacterial meningitis episode were caused by *L. monocytogenes* in 162 patients. Adjunctive dexamethasone 10 mg QID was started with the first dose of antibiotics in 93 of 161 patients (58%) and continued for the full four days in 83 (52%) patients. Different doses, duration or timing of dexamethasone were recorded in 11 patients (7%) and 57 patients (35%) did not receive dexamethasone. The case fatality rate was 51 of 162 (31%) and an unfavourable outcome occurred in 91 of 162 patients (56%). Age and the standard regimen of adjunctive dexamethasone were independent predictors for an unfavourable outcome and mortality. The adjusted odds ratio of dexamethasone treatment for unfavourable outcome was 0.40 (95% confidence interval 0.19–0.81).

**Interpretation:**

Adjunctive dexamethasone is associated with an improved outcome in patients with *L. monocytogenes* meningitis and should not be withheld if *L. monocytogenes* is suspected or detected as causative pathogen.

**Funding:**

10.13039/501100000781European Research Council and 10.13039/501100001826Netherlands Organisation for Health Research and Development

**Research in context**

**Evidence before this study**

We searched ‘*Listeria monocytogenes*’ in combination with “bacterial meningitis” and “dexamethasone” between 1st Jan 2000 and 1st Dec 2022. In 2010, a study from Spain showed dexamethasone was associated with lower mortality (45% vs 67%, p = 0.04) in 43 *L. monocytogenes* meningitis patients, while in 2013 a study of 92 patients in the Netherlands showed dexamethasone treatment was not associated with outcome (odds ratio [OR] 1.46 (95% confidence interval [CI] 0.57–3.73), and in 2014 a Spanish study of 59 patients suggested a lower rate of neurological sequelae in survivors treated with dexamethasone but no effect on mortality. Finally, in 2017 a French nationwide study of 252 patients with neurolisteriosis showed 13% were treated with dexamethasone within 24 h of treatment start, and dexamethasone was associated with poor outcome (OR 4.58 [95% CI 1.50–13.98]).

**Added value of this study**

Our current findings shows that 53% of 162 *L. monocytogenes* meningitis patients received adjunctive dexamethasone 10 mg QID for 4 days which was started with the first dose of antibiotics. Characteristics of patients receiving dexamethasone were comparable to those not receiving dexamethasone according to standard protocol. Treatment with this standard dose of adjunctive dexamethasone was a predictor for a favorable outcome, after correction for age, score on the Glasgow Coma Scale and ineffective initial antibiotic treatment with an Odds ratio of 0.40 (95% confidence interval 0.19–0.81).

**Implications of all the available evidence**

Our results provide evidence that adjunctive dexamethasone is of benefit in patients with *L. monocytogenes* meningitis when given with the first dose of antibiotics in a dose of 10 mg QID for four days. The previously suggested harm of dexamethasone can be explained by different timing of treatment and a potential selection bias. Based on all the available evidence in studies on bacterial meningitis, all patients with community-acquired bacterial meningitis should be treated with a full 4-day course of dexamethasone, independent of the patient's risk factors, comorbidities and the (suspected) pathogen.

## Introduction

Randomized controlled trials, meta-analyses and implementation studies showed that adjunctive dexamethasone treatment improves outcome in bacterial meningitis.^1^, ^2^, ^3^, ^4^ Guidelines therefore advise to start adjunctive dexamethasone treatment in patients with suspected community-acquired bacterial meningitis.^5^^,^^6^ However, guidelines also advise to withhold dexamethasone if *Listeria monocytogenes* is suspected as causative pathogen and to stop dexamethasone treatment if *L. monocytogenes* is identified.^7^ The rationale of this advice is mainly based on results of the MONALISA study, a French nationwide prospective cohort study.^8^ In this study, adjunctive dexamethasone was associated with increased mortality in 252 patients with neurolisteriosis (48% vs 27%). This study stated that 13% of patients with neurolisteriosis received dexamethasone and this treatment was started within 24 h after admission. However, it remained unclear whether in these patients, dexamethasone was administered upon start of antibiotic treatment or later following clinical deterioration. Other cohort studies on *L. monocytogenes* meningitis, showed no detrimental effect of dexamethasone or even suggested benefit.^9^, ^10^, ^11^

In 2006, we started a prospective cohort study to identify the interaction between host and pathogen factors controlling occurrence and outcome of bacterial meningitis (MeninGene).^3^^,^^12^^,^^13^ In 2013 we compared dexamethasone treatment and outcome in 62 *L. monocytogenes* meningitis patients included in this cohort between 2006 and 2012, to 30 patients included between 1998 and 2002 in a previous cohort study and found no association of dexamethasone with outcome.^14^ The number of *L. monocytogenes* meningitis episodes in this cohort has substantially increased over the years, prompting us to re-evaluate the association of dexamethasone with outcome. Here we report data from this study concerning the clinical characteristics, treatment and outcome of adults with *L. monocytogenes* meningitis.

## Methods

### Study population

We identiﬁed adults (patients older than age 16 years) who had bacterial meningitis in the Netherlands between January 1st 2006 and July 1st 2022, and who were listed in the database of the Netherlands Reference Laboratory for Bacterial Meningitis. This laboratory receives cerebrospinal ﬂuid and blood samples from roughly 85% of all patients with bacterial meningitis in the Netherlands (population 17 million). The laboratory provided daily updates of the names of hospitals where patients with bacterial meningitis have been admitted in the preceding 2–6 days, and the names of the attending physicians, usually neurologists. Physicians were informed about the study by telephone. Physicians could also contact investigators at any time to include patients, without preceding report of the reference laboratory. Subsequently, patients or their legal representatives received written information about the study and were asked to give written informed consent for participation. Patients with hospital-acquired meningitis, meningitis following neurotrauma or neurosurgery, and those with a neurosurgical device were excluded.^15^ The study was approved by the Medical Ethics Committee of the Academic Medical Center, University of Amsterdam, the Netherlands.

### Procedures

Clinical data were collected in an online case-record form containing data on clinical characteristics, results of ancillary investigations, antibiotic treatment, dexamethasone treatment and outcome. Patients were considered to be immunocompromised if they had a medical history of diabetes mellitus, cancer, HIV, splenectomy or alcoholism, or if they used immunosuppressive medication. Dexamethasone treatment was considered to be according to protocol when started with the first dose of antibiotics or within 4 h of initiation of antibiotic treatment, in a dose of 10 mg QID for four days. Any other timing, dose or schedule was considered not to be according to protocol, with the exception of patients who died within 4 days and were treated up to their death with the standard protocol. Outcome was scored at hospital discharge according to the Glasgow Outcome Scale (GOS), with scores varying from 1 (death) to 5 (good recovery).^16^ We defined a favourable outcome as a GOS score of 5, and an unfavourable outcome was defined as a score of 1–4. Patients were considered to have *L. monocytogenes* meningitis if they had either a positive CSF culture for the pathogen, or a positive blood culture combined with CSF abnormalities typical for bacterial meningitis, defined as a CSF glucose concentration <340 mg/L, CSF: blood glucose ratio <0.23, protein concentration >2200 mg/L, white cell count >2000 cells/mm^3^, or >1180 polymorphonuclear leucocytes/mm^3^.^17^

### Statistical analysis

Categorical variables are expressed as counts (percentage) and we compared frequency distributions with the Fisher exact test. Continuous variables are expressed as median (interquartile range - IQR). As the continuous data were not normally distributed, we used the Mann–Whitney U or Kruskall–Wallis test to identify differences between groups for continuous variables, and chi^2^ or Fisher exact test to compare categorical outcomes. We compared patients with an unfavourable outcome to those with an favourable outcome, and patients who died to those surviving. We used logistic regression to examine the association between potential predictors of mortality and an unfavourable outcome, and calculated odds ratios (ORs) and 95% confidence intervals (CIs). We included parameters with a p < 0.10 in the univariable analysis to build the multivariable logistics regression model. Because the median number of missing values in the items entered in the multivariable analysis was low (<1%), we did not performed imputation for missing values.

All-cause mortality was analysed as a censored time-to-event variable with Kaplan–Meier methods for patients treated with a standard dose of dexamethasone versus other dexamethasone regimens or no dexamethasone, using the Log–Rank test to determine differences between groups. All statistical tests were 2-tailed, and a P-value of <0.05 was considered statistical significance. All statistical analyses were performed with SPSS version 28.0.

### Role of the funding source

The sponsor of the study had no role in study design, data collection, data analysis, data interpretation, or writing of the report. MCB and DvdB had full access to all the data and had the ﬁnal responsibility for the decision to submit for publication.

## Results

2664 episodes of community-acquired bacterial meningitis in 2578 patients were included, including 162 (6%) patients infected with *L. monocytogenes* of which 62 have been included in a previous study (Supplementary Fig. S1).^14^ The median number of inclusions was 7 per year (interquartile range [IQR 6–11]). The median age of the 162 *L. monocytogenes* meningitis patients was 70 years ([IQR] 61–77) and 56 of 162 (35%) patients were female (Table 1). 97 (60%) of 162 patients used immunosuppressive drugs or had a history of cancer, diabetes, HIV, or alcoholism, and were considered immunocompromised. Seven patients (4%) were younger than 50 years of age and immunocompetent, and therefore lacked risk factors for *L. monocytogenes* infection. Extra-meningeal foci of infection (otitis, sinusitis or pneumonia) were present in 21 (13%) of 159 patients. We did not collect data on preceding gastro-intestinal signs and symptoms, nor on eating habits. Clinical features of meningitis were present in high proportions of patients: headache in 100 of 138 (73%) patients, fever in 128 of 149 (86%), neck stiffness in 87 of 142 (61%), and an altered mental status in 92 of 161 (57%). The classic triad of neck stiffness, fever and altered mental status was present in 50 of 150 (33%) patients.

**Table 1:** Clinical characteristics and laboratory results of patients with *L. monocytogenes* meningitis.

Characteristic^a^	N = 162
**Demographics**	
Age – years^b^	70 (61–77)
Female sex	56/162 (35%)
Immunocompromise	
Diabetes Mellitus	27/160 (17%)
HIV	2/159 (1%)
Immunosuppressive medication	74/161 (46%)
Alcoholism	12/150 (7%)
Active cancer	48/114 (30%)
Extra-meningeal infections	
Pneumonia	17/153 (11%)
Otitis or sinusitis	6/153 (4%)
Antibiotics before admission	16/158 (10%)
Duration of symptoms <24 h	63/156 (40%)
**Symptoms on admission**	
Headache	100/138 (73%)
Nausea	65/134 (49%)
Neck stiffness	87/142 (61%)
Self-reported fever	128/149 (86%)
Seizures	25/154 (16%)
**Physical examination**	
Temperature - °C^c^	39.2 (38.6–39.9)
>38.0 °C	128/150 (85%)
Heart rate - beats/min^d^	95 (82–110)
Systolic blood pressure – mmHg^e^	146 (126–165)
Diastolic blood pressure – mmHg^e^	80 (70–90)
GCS score^f^	13 (10–15)
Altered mental status (<14)	92/161 (58%)
Coma (<8)	16/161 (10%)
Triad of fever, altered mental status and neck stiffness^a^	50/150 (33%)
Aphasia, ataxia, mono- or hemiparesis	36/151 (24%)
**Blood results**^g^	
Thrombocytes - 10ˆ9/L	198 (144–241)
Leukocytes - 10ˆ9/L	12.9 (9.8–16.4)
C-reactive protein - mg/L	88 (40–171)
**CSF chemistry results**^h^	
Leukocytes - cells/mm^3^	879 (357–1759)
<100	11/156 (7%)
100–999	76/156 (49%)
>999	69/156 (44%)
Glucose - mmol/L	2.2 (0.9–3.3)
CSF:blood glucose ratio	0.12 (0.12–0.37)
CSF protein - g/L	2.56 (1.74–3.67)
**Microbiology**	
Positive CSF Gram stain	45/109 (41%)
Positive CSF culture	156/162 (96%)
Positive blood culture	86/135 (64%)

a Data are n/N (%) or median (interquartile range).

b Age was known for all patients.

c Temperature was known in 150 patients.

d Heart rate in 152 patients.

e Blood pressure in 152 patients.

f GCS was known in 161 patients.

g Thrombocyte count was known for 149 patients, leukocyte count in 157 patients, CRP in 155 patients.

h CSF Leukocytes in 156 patients, CSF glucose in 153 patients, CSF to blood Glucose ratio in 135 patients, CSF protein in 153 patients.

A lumbar puncture was performed in all patients. CSF opening pressure was measured in 58 (36%), revealing a median opening pressure of 27 cm H_2_O (IQR 20–38). Median CSF leukocyte count was 879 cells/μl (IQR 357–1759) and CSF protein levels were elevated in 151 of 153 patients (99%; median 2.56 g/L [IQR 1.74–3.67]). Gram stain showed Gram positive rods in 45 of 109 patients (41%) and CSF culture was positive in 156 of 162 (96%). Cranial CT was performed on admission in 137 of 161 patients (85%) showing abnormalities in 42 (30%): old vascular lesions in 17 (12%), recent cerebral infarction in 6 (4%), brain abscess in 6 (4%) and hydrocephalus in 3 (2%). The CT was performed before the lumbar puncture in 107 of 137 patients (78%) and in 41 of 97 (42%) patients antibiotics were started before the CT.

The initial (empirical) antibiotic regimen consisted of amoxicillin and ceftriaxone, in line with the recommendations in the Dutch bacterial meningitis guideline,^18^ in 88 of 158 patients (58%). Other regimens were monotherapy with amoxicillin in 27 (17%), monotherapy with a 3rd generation cephalosporin in 31 patients (20%) or a combination of other antibiotics in 12 (8%). The initial treatment consisted of antibiotics with microbiologically adequate coverage for *L. monocytogenes* in 128 of 161 patients (80%). Out of 33 patients with inadequate antibiotic coverage 21 were initially diagnosed with another infection (14% of all patients): of which 14 were clinically diagnosed by the treating physician with sepsis (67%), 5 pneumonia (24%), and one with Covid-19 infection and with hepatitis (4%). The additional 12 patients were admitted with the diagnosis of bacterial meningitis but treated with third generation cephalosporin monotherapy. Of 7 patients under 50 years old without risk factors for *L. monocytogenes*, 5 were treated with amoxicillin and ceftriaxone and 2 received monotherapy with a 3rd generation cephalosporin. After identification of *L. monocytogenes* treatment was switched to amoxicillin in 154 of 161 (95%) patients. Three of 7 patients who were not treated with amoxicillin died before *L. monocytogenes* was cultured, 2 were treated with gentamicin and 2 with cotrimoxazole because of amoxicillin allergy. The duration of amoxicillin treatment was known for 108 of 111 (97%) surviving patients with a median of 21 days (IQR 19–21). Amoxicillin treatment shorter than 14 days occurred in 10 patients which was due to adverse reactions in all. The daily amoxicillin dose was recorded in 140 of 154 (91%) patients and was 12 g in all, mostly 2 g given every 4 h. Antibiotic treatment included gentamicin in 22 of 161 (14%) patients and cotrimoxazole in 12 of 161 (7%).

Adjunctive dexamethasone treatment was started according to guideline recommendations (10 mg QID for 4 days, started with antibiotics)^5^^,^^6^^,^^19^ in 93 of 161 (58%) patients. In 83 of these patients (89%) the full course of 4 days dexamethasone treatment was given. In the other 10 patients, dexamethasone was discontinued before the end of the 4 days, because *L. monocytogenes* had been cultured. In 11 patients (7%) dexamethasone was given in a different dose, timing or duration and 57 (35%) patients did not receive dexamethasone. Presenting clinical characteristics of patients in whom dexamethasone was started with the first dose of antibiotics were similar to those who received no dexamethasone or another dose or timing, except for higher rates of neck stiffness for those receiving dexamethasone (61 of 86 [71%] vs. 25 of 55 [45%], p = 0.002; Supplementary Table S1), with no difference in blood or CSF parameters of inflammation. Inadequate antibiotic coverage was associated with not receiving dexamethasone treatment; 11 of 31 (35%) patients receiving microbiologically inadequate antibiotic treatment were started on dexamethasone 10 mg together with the first dose of antibiotics vs. 78 of 127 (61%) patients with adequate initial antibiotic treatment (p = 0.01). The antibiotic regimen included amoxicillin for 82 of 83 (99%) patients receiving the standard dexamethasone regimen and for 72 of 78 (92%) in those not receiving dexamethasone according to protocol (p = 0.06; Supplementary Table S2).

**Fig. 1:**
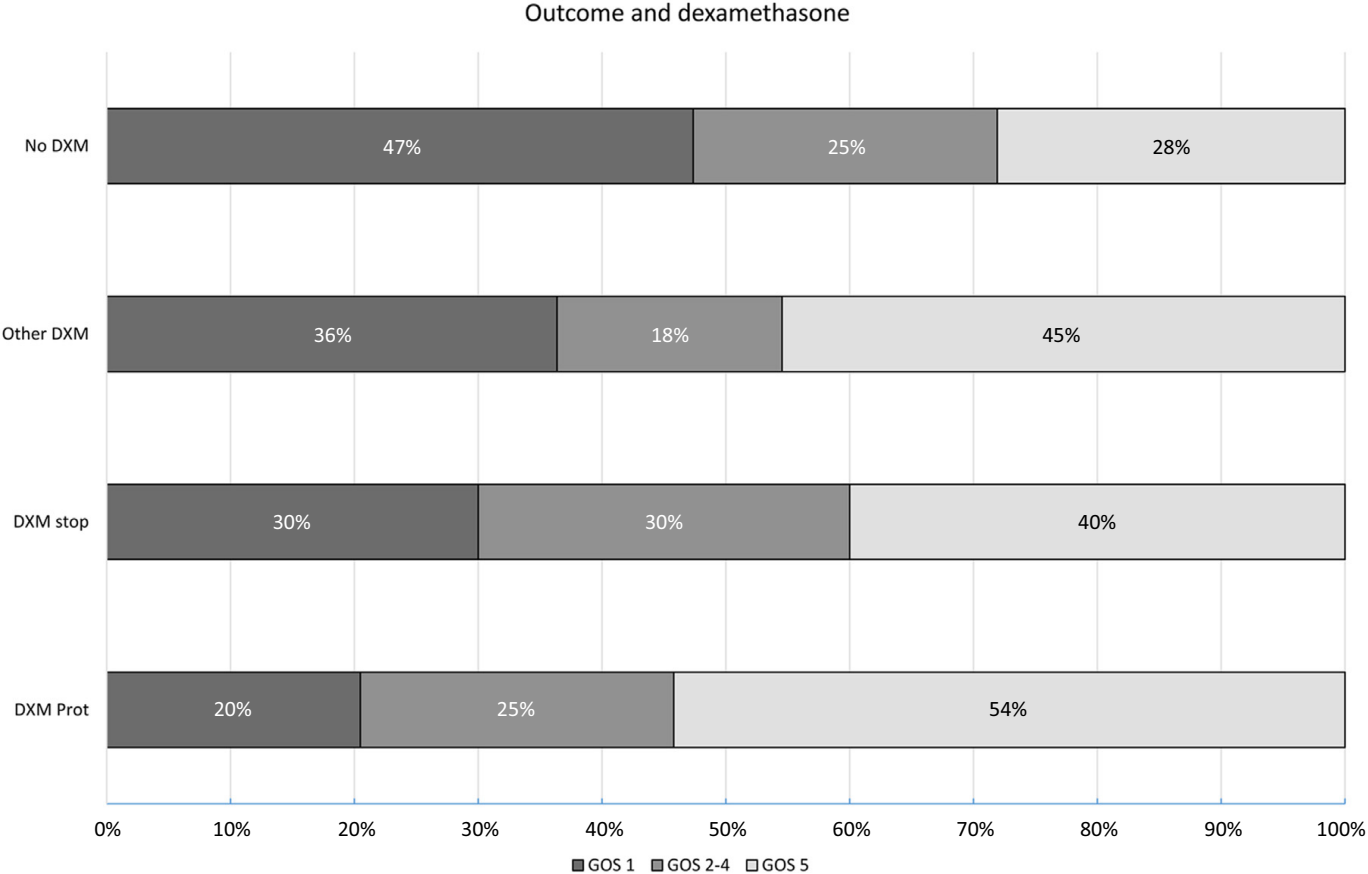
Outcome on Glasgow Outcome Scale score in relation to dexamethasone treatment group. ‘No DXM’ indicates no dexamethasone treatment, ‘DXM stop’ indicates the physician started according to protocol but stopped after listeria was identified, ‘Other DXM’ indicates any other dose, duration or timing of adjunctive dexamethasone treatment than according to standard protocol, and ‘DXM Prot’ indicates 10 mg QID for 4 days started with first dose of antibiotics or <4 h after start of antibiotic treatment.

**Fig. 2:**
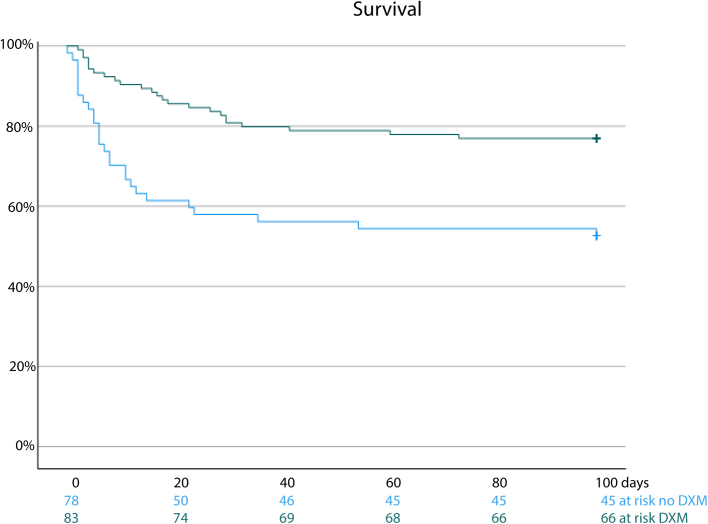
Kaplan-Meijer estimates of survival for listeria meningitis patients receiving standard dexamethasone treatment and those receiving no dexamethasone or a non-standard regimen (log-Rank test P < 0.001).

Complications occurred in a high proportion of patients (Table 2): 22 of 151 (15%) patients developed circulatory shock, 46 of 155 (30%) respiratory failure, 26 of 142 (18%) focal neurologic deficits, 25 of 154 (16%) seizures, 9 of 147 (6%) cerebral infarction, and 7 of 147 (5%) cerebral abscesses. Of the 20 patients who were initially admitted with another diagnosis than meningitis, clinical deterioration consisting of an altered mental status or neurological deficits eventually prompted a lumber puncture, leading to the diagnosis of *L. monocytogenes* meningitis. A total of 51 of 162 (32%) patients died, 8 were severely disabled (5%; GOS score 3), 32 were moderately disabled (20%; GOS score 4) and 71 patients had a good recovery (44%). Outcome in the 20 patients initially misdiagnosed and treated with microbiologically inadequate antibiotics was unfavourable in 16 (80%), of whom 11 (55%) died. Cognitive defects were reported upon discharge in 21 of 93 surviving patients (23%). The rate of unfavourable outcome was 72% in patients who did not receive dexamethasone, 46% in those receiving the adjunctive dexamethasone regimen, 60% in those in whom dexamethasone was discontinued after identification of *L. monocytogenes* meningitis and 55% in patients who received a different regimen of dexamethasone (Fig. 1). Adjunctive dexamethasone according to standard protocol was associated with improved survival in a Kaplan–Mayer estimate (log Rank test p < 0.001, Fig. 2).

**Table 2:** Treatment, complications and outcome in *L. monocytogenes* meningitis.

Characteristic^a^	N = 162
**Initial antibiotic treatment**	
3rd gen cephalosporin	31/161 (20%)
Amoxicillin	27/161 (17%)
3rd gen ceph + amoxicillin	91/161 (57%)
Other	12/161 (8%)
**Final antibiotic treatment**	
Amoxicillin	154/161 (95%)
Duration of amoxicillin treatment in survivors (days)^b^	21 (19–21)
Regimen incl. gentamicin	22/161 (14%)
**Dexamethasone**	
4 days 10 mg QID with or <4 h of first dose of antibiotics	83/161 (52%)
Stopped after *Listeria* cultured	10/161 (6%)
Other dose or timing	11/161 (7%)
No dexamethasone	57/161 (35%)
**Complications**	
Pneumonia	27/152 (18%)
Transfer to ICU	63/148 (43%)
Mechanical ventilation	29/154 (19%)
Seizures	25/154 (16%)
Cerebrovascular accident	9/147 (6%)
Hydrocephalus	11/145 (7%)
**GOS score**	
1 (death)	51/162 (31%)
2 (vegetative state)	0
3 (severe disability)	8/162 (5%)
4 (moderate disability)	32/162 (20%)
5 (mild or no disability)	71/162 (44%)
**Sequelae**	
Cognitive impairment	21/93 (23%)
Hearing impairment	3/93 (3%)
Motor deficit	5/93 (5%)
Cranial nerve palsy	6/93 (6%)

a Data are n/N (%) of median (interquartile range).

b Duration of treatment known in 108 of 111 surviving patients.

Univariable analysis showed increasing age, lower admission score on the Glasgow Coma Scale, inadequate initial antibiotic coverage of *L. monocytogenes* and not receiving dexamethasone treatment according to the guideline to be associated with unfavourable outcome (Table 3). An antibiotic regimen which included gentamicin did not influence outcome: 14 of 22 (63%) patients treated with gentamicin had an unfavourable outcome compared to 76 of 139 (55%) who not treated with gentamicin (p = 0.49). Also after correction for age, GCS score and dexamethasone, treatment with gentamicin was not associated with outcome. Time period analyses showed no differences between 2006 and 2011, 2012–2016 and 2017–2022 with respect to clinical characteristics or outcome. Patients included between 2017 and 2022 were less likely to receive gentamicin compared to 2006–2011 and 2012–2016, other treatment characteristics including dexamethasone treatment were comparable over time (Supplementary Table S3). In a multivariable analysis age (OR per year increase 1.04; 95% CI 1.01-1.07) and standard regimen dexamethasone treatment (OR 0.40; 95%CI 0.19–0.81) were independent predictors for unfavourable outcome. In a sensitivity analysis, excluding the 20 patients initially misdiagnosed, the predictive effect of a standard regimen dexamethasone treatment remained robust in the multivariable analysis (OR 0.47; 95% CI 0.22–1.00). A multivariable analysis on mortality showed increasing age and not receiving dexamethasone treatment according to protocol were associated with an increased risk of death, after correction for GCS score and effective antibiotic treatment (Table 4).

**Table 3:** Results of univariable and multivariable analysis of risk factors for unfavourable outcome in *L. monocytogenes* meningitis.

Variable	Univariable analysis		Multivariable analysis	
	OR (95% CI)	*P*-value	OR (95% CI)	*P*-value
Age (per year increase)	1.04 (1.01-1.06)	0.002	1.04 (1.01-1.06)	0.007
Male sex	1.19 (0.61–2.29)	0.61		
Immunocompromised state^a^	0.95 (0.50–1.79)	0.88		
Glasgow Coma Scale (per point increase)	0.90 (0.79–1.01)	0.07	0.91 (0.80–1.06)	0.14
C-reactive protein (per 10 mg/L)	1.02 (0.98–1.05)	0.31		
CSF leukocyte count (per 100 cells/mm^3^)	1.00 (0.97–1.02)	0.67		
Adequate initial antibiotic regimen^a^	0.33 (0.14–0.79)	0.01	0.44 (0.17–1.09)	0.08
Dexamethasone 10 mg QID 4 days^a^^,^^b^	0.40 (0.21–0.76)	0.005	0.40 (0.19–0.81)	0.017

a Reference categories: no immunocompromised state, no adequate initial antibiotic regimen and no dexamethasone treatment according to standard protocol.

b Full course for 4 days started with 1st dose of antibiotics.

**Table 4:** Results of univariable and multivariable analysis of risk factors for mortality in *L. monocytogenes* meningitis.

Variable	Univariable analysis		Multivariable analysis	
	OR (95% CI)	*P*-value	OR (95% CI)	*P*-value
Age (per year increase)	1.05 (1.02–1.09)	0.003	1.04 (1.01-1.08)	0.010
Male sex	0.92 (0.46–1.86)	0.82		
Immunocompromised state^a^	1.91 (0.60–2.36)	0.61		
Glasgow Coma Scale (per point increase)	0.88 (0.78–0.99)	0.045	0.88 (0.77–1.01)	0.06
C-reactive protein (per 10 mg/L)	1.02 (0.99–1.06)	0.22		
CSF leukocyte count (per 100 cells/mm^3^)	1.01 (0.98–1.03)	0.55		
Adequate initial antibiotic regimen^a^	0.40 (0.18–0.88)	0.02	0.12 (0.21–1.19)	0.11
Dexamethasone 10 mg QID 4 days^a^^,^^b^	0.33 (0.17–0.67)	0.002	0.40 (0.19–0.84)	0.016

a Reference categories: no immunocompromised state, no adequate initial antibiotic regimen and no dexamethasone treatment according to standard protocol.

b Full course for 4 days started with 1st dose of antibiotics.

## Discussion

This study shows that adjunctive dexamethasone is associated with a reduced risk for unfavourable outcome and death in adults with community-acquired *L. monocytogenes* meningitis. Although our study is a prospective cohort study and not a randomized controlled trial, it presents compelling evidence that adjunctive dexamethasone treatment is not harmful in *L. monocytogenes* meningitis. A randomized controlled trial showed that early treatment with dexamethasone improves the outcome in adults with acute bacterial meningitis.^1^ The effect of dexamethasone was most apparent in the group of patients with pneumococcal meningitis and those most severely ill.^1^ Meta-analyses showed that dexamethasone is beneficial for children beyond the neonatal age and adults, while subgroup analyses showed strongest effects in pneumococcal meningitis and meningitis due to *Haemophilus influenzae*.^2^^,^^20^ Implementation studies showed that the introduction of dexamethasone has led to improved outcome of bacterial meningitis for pneumococcal meningitis on a nationwide level, but also for non-pneumococcal meningitis.^3^^,^^21^ Our data contrast with the French nationwide cohort study on neurolisteriosis.^8^ This study involving 252 patients from the period 2009–2013 reported reduced survival in patients with neurolisteriosis treated with adjunctive dexamethasone, which has resulted to guideline recommendations to withhold dexamethasone in patients with neurolisteriosis.^5^^,^^6^

The discrepancy between our study and that of the French cohort may be explained in several ways. First, in the French cohort 13% of patients received adjunctive dexamethasone as compared to 58% in our cohort, providing us with larger numbers per group with and without dexamethasone, enabling us to better identify differences between groups. Second, results of the French study may well be confounded by indication. Data on the severity of patients and timing of dexamethasone (other than within 24 h after admission) was not provided in the French study, but it may well be that adjunctive dexamethasone was used in the most severely ill patients. In our cohort we show clinical characteristics between patients who did and those who did not receive dexamethasone were similar, except for neck stiffness. Third, in the Netherlands the majority of those treated with dexamethasone received it together with the antibiotics, while detailed data on timing of dexamethasone is lacking in the French cohort. It has been shown that dexamethasone is most effective in reducing the inflammatory response when given early in the disease course as antibiotic treatment causes rapid bacteriolysis and subsequently high concentrations of inflammatory bacterial fragments.^22^^,^^23^ Our cohort study included detailed information on clinical features, timing and dose of dexamethasone regimen used, allowing more detailed analyses on the association between clinical severity, use and timing of dexamethasone, and outcome. The beneficial effect of dexamethasone remained robust in sensitivity analyses excluding those patients initially misdiagnosed and undertreated.

Our study shows that a considerable proportion of patients with *L. monocytogenes* meningitis do not receive adequate initial antimicrobial treatment. Patients were either not recognized as having meningitis or treated with third generation cephalosporins, an antimicrobial agent without activity against *L. monocytogenes*. Although previous research showed that patients with meningitis due to *L. monocytogenes* do not present with atypical clinical features,^14^^,^^24^ about 12% of patients was initially diagnosed with infections elsewhere in the body, mainly sepsis. These patients were often treated with suboptimal antimicrobial therapy. Clinical deterioration during clinical course eventually prompted a lumbar puncture, leading to the diagnosis of listerial meningitis. According to IDSA and ESCMID bacterial meningitis treatment guidelines, adult neurolisteriolis should be treated with IV beta-lactam antibiotics during at least 21-days.^5^^,^^14^^,^^25^ Because the causative pathogen will not be known when starting antibiotics, empirical treatment of adults with risk factors for neurolisteriosis (either older than 50 years, or immunocompromised status) should include therapy covering *L. monocytogenes*. According to the Dutch bacterial meningitis guideline, all patients are advised to be treated with ceftriaxone and amoxicillin irrespective of risk factors for listeriosis.^19^ Overall adherence to this guideline was however low in *L. monocytogenes* meningitis patients (57%). Treatment according to the guideline would have covered the 7 patients (4%) in our cohort that were younger than 50 years of age and immunocompetent.^19^ Naturally, empirical treatment should be modified (stepped down) as soon as the causative pathogen, and the antimicrobial sensitivity results of the causative organism are known.

Even though randomized controlled trials on adjunctive dexamethasone in bacterial meningitis showed benefit for all patients with bacterial meningitis,^2^ the lack of proven efficacy of dexamethasone in bacterial meningitis due to other pathogens than *S. pneumoniae* results in hesitancy to treat patients with dexamethasone.^6^, ^25^ The 2016 UK guideline on bacterial meningitis urged to stop dexamethasone if another pathogen than *S. pneumoniae* is detected or probable.^6^ A study describing clinical practice in the UK and Ireland in 2017 showed that only 21% of 1471 bacterial meningitis patients received dexamethasone.^26^ The reason for this hesitancy appears to be a perceived risk of dexamethasone treatment, even though no detrimental effect has been identified in subgroups of bacterial meningitis patients using immunosuppressive medication, those with diabetes mellitus or cancer, or those with *Neisseria meningitidis* as causative pathogen.^27^, ^28^, ^29^, ^30^ Now we show that in the only subgroup of bacterial meningitis patients in whom dexamethasone has been suggested to cause harm, it is associated with a better outcome. In our opinion, guidelines should advise to treat all patients with community-acquired bacterial meningitis with a full 4-day course of dexamethasone, independent of the patient's risk factors, comorbidities and the (suspected) pathogen.

Our study has several limitations. First, analysing treatment effects from observational data is prone to selection bias. Our cohort with extensive clinical data allowed multivariable analysis showing dexamethasone to remain a strong independent predictor of outcome. The detected effect size of dexamethasone is similar to the identified effect in randomized controlled trials and implementation studies of adjunctive dexamethasone in bacterial meningitis.^1^, ^2^, ^3^ It is important to realize with the detected beneficial effect of dexamethasone, it can be considered extremely unlikely that dexamethasone would negatively influence unfavourable outcome and death. Nevertheless, a randomized controlled trial would be preferable to definitively determine whether dexamethasone has a beneficial effect on outcome in *L. monocytogenes* meningitis. However, this is very unlikely to be feasible because of the low incidence. Second, all patients underwent lumbar puncture and CSF analysis. This may have result in selection bias: we may have missed patients with listerial meningitis who have received antibiotic treatment for an initially wrongful diagnosis of respiratory, urinary tract or bowel infection, in whom no lumbar puncture was performed, or the CSF had been sterilized due to the antibiotic treatment. This could result in a selection towards more severely affected patients, but is unlikely to influence the analysis of dexamethasone treatment. Thirdly, the association between dexamethasone and outcome might be confounded by the misdiagnosis of *L. monocytogenes* meningitis. Patients who were misdiagnosed were less likely to be treated with dexamethasone and had a high case fatality rate (55%). To overcome this potential bias we performed a sensitivity analysis by excluding those patients initially misdiagnosed and undertreated, which showed the beneficial effect of dexamethasone remained robust.

In conclusion, we show that adjunctive dexamethasone was associated with a substantially improved outcome in adults with *L. monocytogenes* meningitis in our nationwide cohort study. We identified that a considerable proportion of patients with neurolisteriosis was initially misdiagnosed and undertreated, indicating that physicians should have a low threshold for cerebrospinal fluid examination to rule the possibility of meningitis in patients with sepsis and treat patients with empiric antimicrobial treatment covering *L. monocytogenes* if risk factors are present.

## Contributors

MCB and DvdB designed the study, collected, analysed, and interpreted data, and wrote the report.

## Data sharing statement

Individual participant data underlying the results reported in this article will be available on reasonable request, after de-identification. Proposals should be directed to the corresponding author. A data access agreement should be signed by data requestors.

## Declaration of interests

MCB is supported by research grants of the 10.13039/501100000781European Research Council, 10.13039/501100001826Netherlands Organisation for Health Research and Development and stichting de Merel. MCB is supported for travel and attending the ECCMID by the European Society of Clinical Microbiology and Infectious Disease (ESCMID). MCB participates in the Trial Steering Committee of the ENCEPH-UK trial and chairs the ESCMID study group on infections of the brain. DvdB is supported by a research grant of the Netherlands Organization for Health Research and Development. DvdB participates in the Trial Steering Committee of the DEX-ENCEPH trial.
