# Supplementary material for: Adjunctive dexamethasone treatment in adults with listeria monocytogenes meningitis: a prospective nationwide cohort study
Source: eClinicalMedicine. 2023 Mar 24;58:101922. doi: 10.1016/j.eclinm.2023.101922 (PMC10050789; doi:10.1016/j.eclinm.2023.101922)
Supplement: Supplementary Fig. S1 and Tables S1–S3 [file mmc1.docx]

**SUPPLEMENTARY MATERIAL**

**Supplementary Table 1.** Clinical characteristics and laboratory results of *L. monocytogenes* meningitis patients initially treated with dexamethasone 10mg versus those not started on dexamethasone or receiving a different dose or timing of dexamethasone.

| **Characteristic^a^** | **DXM started (N=93)** | **DXM not started or in a non-standard dose or timing (N=68)** | **P-value^b^** |
| --- | --- | --- | --- |
| **Demographics** |  |  |  |
| Age – years^c^ | 69 (61-76) | 73 (63-79) | 0·16 |
| Female sex | 30/93 (32%) | 25/68 (37%) | 0·62 |
| **Predisposing factors** |  |  |  |
| Immunocompromise | 55/93 (59%) | 50/68 (74%) | 0·07 |
| Antibiotics before admission | 8/92 (9%) | 7/65 (11%) | 0·78 |
| Pneumonia | 6/86 (7%) | 11/66 (17%) | 0·07 |
| Otitis or sinusitis | 2/87 (2%) | 5/66 (8%) | 0·24 |
| **Symptoms and signs on admission** |  |  |  |
| Duration of symptoms < 24 hours | 48/90 (53%) | 21/65 (32%) | 0·10 |
| Headache | 62/81 (77%) | 37/56 (66%) | 0·24 |
| Neck stiffness | 61/86 (71%) | 25/55 (45%) | **0·002** |
| Temperature - °C^d^ | 39·5 (38·7-40·0) | 39·1 (38·3-39·7) | 0·07 |
| Heart rate - beats/min^e^ | 95 (82-108) | 99 (87-110) | 0·36 |
| GCS score^f^ | 13 (10-14) | 13 (10-15) | 0·99 |
| Meningitis triad^g^ | 38/88 (43%) | 12/59 (20%) | **0·004** |
| Seizures | 4/90 (4%) | 3/65 (5%) | >0·99 |
| Focal neurologic deficits | 25/89 (28%) | 11/61 (18%) | 0·18 |
| **Blood results**^h^ |  |  |  |
| Leukocytes - 10^9/L | 13·2 (10·4-16·4) | 12·6 (8·5-16·5) | 0·60 |
| C-reactive protein - mg/L | 83 (37-186) | 93 (45-174) | 0·49 |
| **CSF chemistry results**^i^ |  |  |  |
| Leukocytes - cells/mm^3^ | 905 (400-1768) | 844 (304-1781) | 0·56 |
| CSF:blood glucose ratio | 0·26 (0·15-0·39) | 0·22 (0·10·-0·36) | 0·21 |
| CSF protein - g/L | 2·4 (1·7-3·6) | 2·6 (1·8-3·8) | 0·78 |
| **Antibiotic treatment** |  |  |  |
| 3^rd^ gen cephalosporin | 11/92 (12%) | 20/68 (29%) | **<0·001** |
| Amoxicillin | 12/92 (13%) | 14/68 (21%) |  |
| 3^rd^ gen ceph + amoxicillin | 67/92 (73%) | 24/68 (35%) |  |
| Other | 2/92 (2%) | 10/68 (15%) |  |
| Antibiotic regimen covering  *L. monocytogenes* | 81/92 (88%) | 46/68 (68%) | **0·002** |

^a^Data are n/N (%) or median (interquartile range), ^b^Difference between groups were calculated with Fisher-exact test or chi^2^ for categorical variables and the student‘s T-test for normally ditributed continuous variables and Mann-Whitney U test for non-normally distributed continuous variables, ^c^Age was known for all patients, ^d^Temperature was known in 149 patients, ^e^Heart rate in 152, ^f^GCS was known in 155 patients, ^g^Neck stiffness, fever and altered mental status, ^h^Leukocyte count in 156, C-reactive protein in 154, ^i^CSF leukocyte count was known in 155 patients, CSF to blood glucose ratio in 134 and CSF protein in 152.

| **Characteristic^a^** | **DXM 10mg QID 4 days (N=83)** | **No DXM or other regimen (N=78)** | **P-value^b^** |
| --- | --- | --- | --- |
| **Antibiotic regimen including** |  |  |  |
| Amoxicillin | 82/83 (99%) | 72/78 (92%)^a^ | 0·06 |
| Gentamycin | 9/82 (11%) | 13/78 (17%) | 0·36 |
| Cotrimoxazole | 7/82 (9%) | 5/78 (6%) | 0·77 |

**Supplementary Table 2.** Antibiotic treatment *L. monocytogenes* meningitis patients treated with dexamethasone 10mg QID for 4 days versus those not started on dexamethasone or receiving a different dose or timing of dexamethasone.

^a^Three patients died prior to identification of *L. monocytogenes*

**Supplementary Table 3.** Clinical characteristics, treatment and outcome *L. monocytogenes* meningitis patients over time per 5 year period.

| **Characteristic^a^** | **2006-2011 (n=59)** | **2012-2016 (n=42)** | **2017-2022 (n=61)** | **p-value^b^** |
| --- | --- | --- | --- | --- |
| **Demographics** |  |  |  |  |
| Age – years^c^ | 69 (61-76) | 68 (61-76) | 71 (61-81) |  |
| Female sex | 21/59 (36%) | 14/42 (33%) | 21/62 (34%) | 0·97 |
| **Predisposing factors** |  |  |  |  |
| Immunocompromise | 33/59 (56%) | 29/42 (69%) | 35/61 (57%) | 0·17 |
| Antibiotics before admission | 6/59 (10%) | 4/41 (10%) | 6/58 (10%) | 0·99 |
| Immunosuppressive medication | 24/59 (41%) | 24/41 (59%) | 26/61 (43%) | 0·17 |
| Active cancer | 10/59 (17%) | 8/41 (20%) | 8/61 (13%) | 0·68 |
| Diabetes mellitus | 9/59 (15%) | 7/40 (18%) | 11/61 (18%) | 0·91 |
| Pneumonia | 5/58 (9%) | 5/37 (12%) | 7/58 (12%) | 0·73 |
| Otitis or sinusitis | 2/58 (3%) | 1/36 (2%) | 4/59 (7%) | 0·58 |
| **Symptoms and signs on admission** |  |  |  |  |
| Duration of symptoms < 24 hours | 22/59 (37%) | 18/39 (43%) | 23/58 (38%) | 0·68 |
| Headache | 43/54 (80%) | 21/32 (66%) | 36/52 (70%) | 0·30 |
| Neck stiffness | 37/57 (66%) | 24/35 (69%) | 26/50 (52%) | 0·23 |
| Temperature - °C^d^ | 39.5 (38.8-40.1) | 39.0 (38-39.6) | 39-1 (38.6-39.9) | 0·11 |
| Heart rate - beats/min^e^ | 100 (87-120) | 93 (84-103) | 95 (81-110) | 0·31 |
| GCS score^f^ | 13 (10-14) | 12 (11-15) | 14 (11-15) | 0·26 |
| Meningitis triad | 18/41 (31%) | 15/36 (42%) | 17/55 (31%) | 0·48 |
| Seizures | 1/59 (2%) | 3/38 (7%) | 3/59 (5%) | 0·34 |
| Focal neurologic deficits | 12/58 (21%) | 14/40 (35%) | 10/53 (20%) | 0·15 |
| **Blood results**^g^ |  |  |  |  |
| Leukocytes - 10^9/L | 13.6 (10.6-17.1) | 12.0 (9.7-15.8) | 12.5 (8.6-16.7) | 0·11 |
| C-reactive protein - mg/L | 115 (54-191) | 110 (47-172) | 61 (26-134) | 0·051 |
| **CSF chemistry results**^h^ |  |  |  |  |
| Leukocytes - cells/mm^3^ | 720 (356-1573) | 758 (296-1698) | 1000 (372-1971) | 0·54 |
| CSF:blood glucose ratio | 0.25 (0.12-0.37) | 0.17 (0.11-0.33) | 0.25 (0.13-0.43) | 0·48 |
| CSF protein - g/L | 2.6 (1.7-3.7) | 2.2 (1.8-3.8) | 2.6 (1.7-3.5) | 0·97 |
| **Initial antibiotic treatment** |  |  |  |  |
| 3^rd^ gen cephalosporin | 13/59 (22%) | 4/42 (10%) | 14/60 (23%) | 0·14^i^ |
| Amoxicillin | 11/59 (22%) | 6/42 (14%) | 10/60 (17%) |  |
| 3^rd^ gen ceph + amoxicillin | 33/59 (56%) | 25/42 (60%) | 33/60 (54%) |  |
| Other | 2/59 (3%) | 7/42 (17%) | 3/60 (5%) |  |
| AB covering *L. monocytogenes* | 46/59 (78%) | 38/42 (91%) | 44/60 (72%) | 0·10 |
| **Total antibiotic regimen** |  |  |  |  |
| Regimen incl. amoxicillin | 58/59 (98%) | 38/42 (91%) | 59/61 (97%) | 0·14 |
| Regimen incl. gentamycin | 11/59 (19%) | 10/42 (24%) | 1/60 (2%) | **0·02** |
| Regimen incl. cotrimoxazole | 5/59 (9%) | 4/42 (10%) | 3/60 (5%) | 0·65 |
| **Dexamethasone treatment** |  |  |  |  |
| 4 days 10mg QID with or <4h   of first dose of antibiotics | 34/59 (58%) | 17/42 (40%) | 32/60 (53%) | 0·27 ^i^ |
| Stopped after *Listeria* cultured | 2/59 (3%) | 3/42 (7%) | 5/60 (8%) |  |
| Other dose or timing | 3/59 (5%) | 6/42 (14%) | 2/60 (3%) |  |
| No dexamethasone | 20/59 (34%) | 16/42 (38%) | 21/60 (35%) |  |
| **GOS score** |  |  |  |  |
| 1 (death) | 20/59 (34% | 15/42 (36%0 | 16/60 (27%) | 0·53^j^ |
| 2 (vegetative state) | 0 | 0 | 0 |  |
| 3 (severe disability) | 5/59 (8%) | 1/42 (2%) | 2/60 (3%) |  |
| 4 (moderate disability) | 9/59 (15%) | 7/42 (17%) | 16/60 (27%) |  |
| 5 (mild or no disability) | 25/59 (42%) | 19/42 (45%) | 27/60 (45%) | 0·96^k^ |

^a^Data are median (interquartile range0 or n/N (%), ^b^Difference between groups were calculated with Fisher-exact test or chi^2^ for categorical variables and Kruskall-Wallis test for continuous variables,^c^Age was known for all patients, ^d^Temperature was known for 59 (2006-2011), 36 (2012-2016) and 55 patients (2017-2022), ^e^Heart rate was known for 59 (2006-2011), 38 (2012-2016) and 55 patients (2017-2022), ^f^GCS score was known for 59 (2006-2011), 41 (2012-2016) and 61 patients (2017-22), ^g^Blood leukocyte count was known for 58 (2006-2011), 40 (2012-2016) and 59 patients (2017-2022), CRP for was known for 59 (2006-2011), 38 (2012-2016) and 58 patients (2017-2022), ^h^CSF leukocyte countr was known for 59 (2006-2011), 40 (2012-2016) and 57 patients (2017-2022), CSF to blood glucose ratio in 53 (2006-2011), 34 (2012-2016) and 48 patients (2017-2022), CSF protein concentration in 58 (2006-2011), 38 (2012-2016) and 57 patients (2017-2022), ^i^Overall chi^2^ test for group differences per time period, ^j^P-value for mortality over time, ^k^P-value for favourable vs unfavourable outcome over time.

Supplementary figure 1. Flowchart of patient inclusion.
